# Neural Correlates of Phrase Rhythm: An EEG Study of Bipartite vs. Rondo Sonata Form

**DOI:** 10.3389/fninf.2017.00029

**Published:** 2017-04-27

**Authors:** Arturo Martínez-Rodrigo, Alicia Fernández-Sotos, José Miguel Latorre, José Moncho-Bogani, Antonio Fernández-Caballero

**Affiliations:** ^1^Departamento de Sistemas Informáticos, Universidad de Castilla-La Mancha, Cuenca, Spain; ^2^Conservatorio Profesional de Música Narciso Yepes, Murcia, Spain; ^3^Departamento de Psicología, Universidad de Castilla-La Mancha, Albacete, Spain; ^4^Departamento de Ciencias Médicas, Universidad de Castilla-La Mancha, Albacete, Spain; ^5^Departamento de Sistemas Informáticos, Universidad de Castilla-La Mancha, Albacete, Spain

**Keywords:** phrase rhythm, brain activity, electroencephalography (EEG), brain–computer interface (BCI), music-evoked stimuli

## Abstract

This paper introduces the neural correlates of phrase rhythm. In short, phrase rhythm is the rhythmic aspect of phrase construction and the relationships between phrases. For the sake of establishing the neural correlates, a musical experiment has been designed to induce music-evoked stimuli related to phrase rhythm. Brain activity is monitored through electroencephalography (EEG) by using a brain–computer interface. The power spectral value of each EEG channel is estimated to obtain how power variance distributes as a function of frequency. Our experiment shows statistical differences in *theta* and *alpha* bands in the phrase rhythm variations of two classical sonatas, one in bipartite form and the other in rondo form.

## 1 Introduction

Our interest in music-evoked emotions is linked to finding solutions able to improve the quality of life and care of aging adults who can or want to keep living at home. The approach uses advanced tools and techniques of Information Technology and Communications supplemented with expert knowledge based on experimental techniques from psychology, neurobiology, and music about the regulation of emotions (Fernández-Caballero et al., 2014, 2016; Castillo et al., 2016). More concretely, this paper attempts to show the neural correlates of phrase rhythm which cause changes in the emotional state of a music listener through conducting an experiment (similar to Fernández-Sotos et al. (2015, 2016)). Neural correlates of emotional responses have been explored by a number of researchers (Schmidt and Trainor, 2001). The potential of music to evoke emotions makes music a valuable tool for the investigation of emotions (Koelsch, 2014). There are currently only a few studies on the psychophysiology of the perception of the vertical harmonic structure of music by humans (Maslennikova et al., 2015). The neuronal correlates of the hierarchy of musical tones and their ratios at the cortical level in humans are generally studied using evoked potential methods and functional magnetic resonance imaging (fMRI) (Minato et al., 2009; Koelsch, 2011). This paper provides one-step forward toward understanding the neural correlates of phrase rhythm. For this aim, an approach based on monitoring electroencephalography (EEG) signals is fully introduced.

Phrase rhythm is, in its most basic sense, the rhythm articulated by a series of phrases. In music and music theory, a musical phrase is a unit of musical meter that has a complete musical sense of its own, built from figures, motifs, and cells and combining to form melodies, periods, and larger sections (Nattiez, 1990), or the length in which a singer or instrumentalist can play in one breath. White (1976) defines a phrase as “the smallest musical unit that conveys a more or less complete musical thought.” Let us highlight that phrase rhythm is the rhythmic aspect of phrase construction and the relationships between phrases (Rothstein, 1989). For the composer of a piece, it is important that the listener relates different moments of a play and looks forward to new musical resources. In this way, the composer uses the principles of repetition and contrast. Ideas and parts heard before (phrases equal to each other) are used in the repetition, while with the contrast, the composer proposes new ideas to create expectation. These are precisely the aspects studied to find the neural correlates of phrase rhythm.

The rest of the paper is as described next. Section 2 introduces the materials and methods employed in the experimentation. The musical experiment that is designed to induce music-evoked stimuli is explained first. Two classical music pieces with sufficient differences in their phrase structure are used to search the neural correlates of phrase rhythm. These are the first movement of the sonatina in bipartite sonata form by Wolfgang Amadeus Mozart named “Sonatina for clarinet and piano in Bb Major” (Mozart, 1959), and the first movement of James Hook’s “Sonata No. 1 for trumpet and piano in Eb Major” in rondo form (Hook, 2003). This section also includes a brief description of the Emotiv Epoc+ brain–computer interface (BCI) used to acquire the EEG signals. Moreover, the study carried out by means of the musical experiment is described. Also, the four stages used to process the BCI data and to validate the proposal are described in detail. These are signal acquisition, data preprocessing, feature extraction, and classification and statistical analysis. Afterward, Section 3 describes the most outstanding results of the study carried out, showing the first evidences related to the neural correlates of phrase rhythm. Finally, Section 4 provides some discussion and conclusions.

## 2 Materials and Methods

### 2.1 Description of the Experimentation

The experimentation is carried out in an especially organized room, where each participant is placed in front of a computer. A total of twenty healthy subjects (60% male, age 35.22 ± 9.34) participate in the experiment. Two different classical music pieces (Mozart and Hook), both with a 2-min duration, are presented to each participant. During the experiment, electroencephalogram recordings were acquired using an Emotiv EPOC+ headset, transforming brain activity into electrical signals that are amplified and digitized for further processing. In fact, the Emotiv Epoc+ is the EEG data acquisition means used in this study. It is a helmet whose main virtue is its easy setup and use, requiring a few minutes of preparation by the user who will use it. The helmet has 14 channels for detecting EEG signals and two references, taking readings of brain activity at a frequency of 128 Hz. The data transmission from the helmet to the computer is performed wireless. Emotiv Epoc+ also provides a series of facilities, as it includes a dedicated software for direct access to the data acquired by the helmet. In addition, you can access the status of signal quality of the electrodes’ signals and the recorded data.

**Figure 1:**
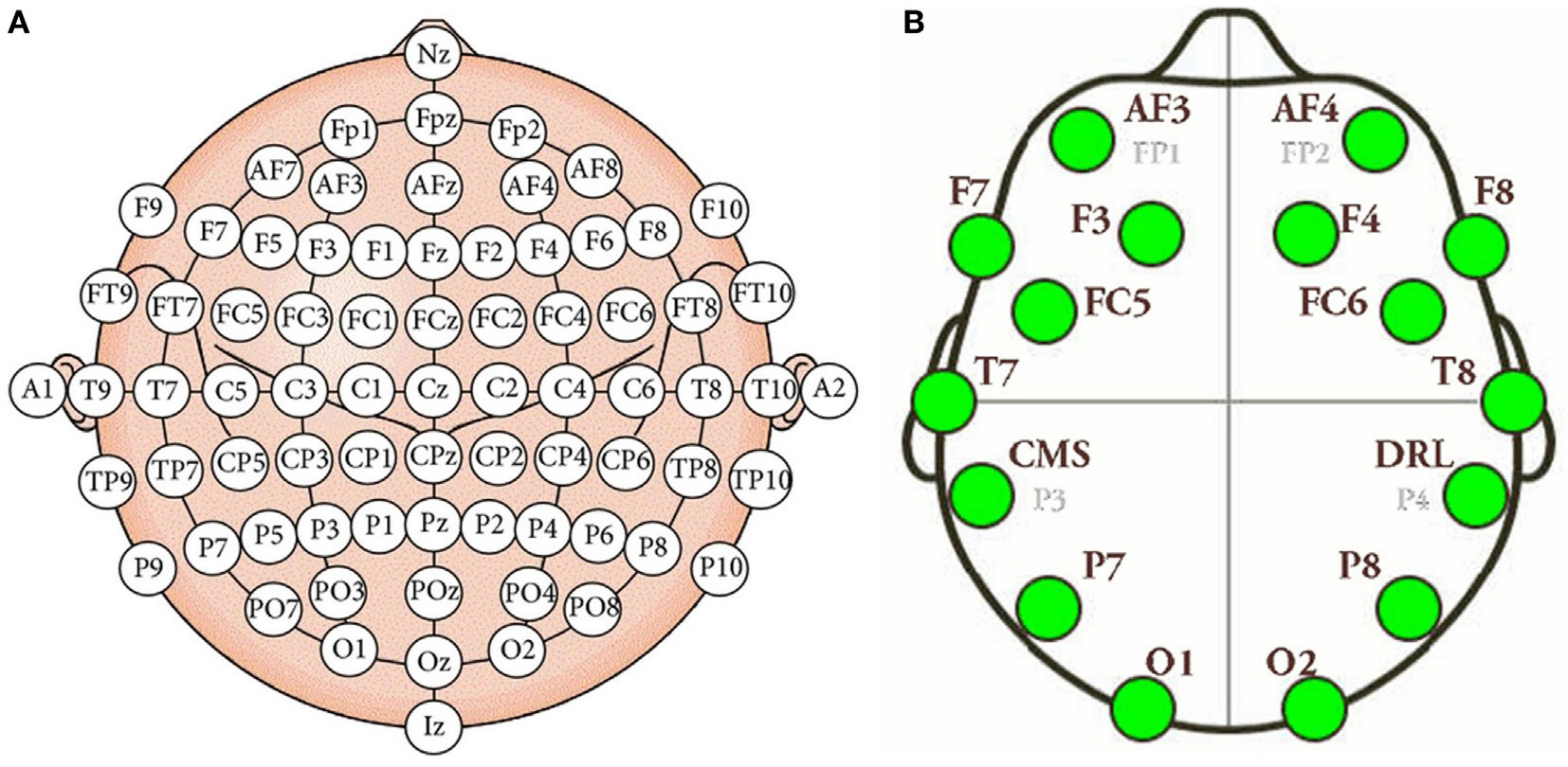
**(A)** International 10–20 system of electrode placement. **(B)** Emotiv Epoc+ electrode placement.

Electroencephalography (EEG) measures the electrical activity of the brain using electrodes attached to the scalp (Tonoyan et al., 2016). The electrodes are activated by the flow of electrical currents during synaptic excitation of the dendrites in neurons. On the other hand, a brain–computer interface (BCI) is a man–machine interaction system capable of capturing, processing, and interpreting brain waves. The interest in these devices in developers and researchers for technological purposes is triggered by the rapid and continuous development of low-cost hardware and software systems supporting multichannel analysis in real time. Mostly all EEG devices roughly consist of the same parties, although each manufacturer provides certain characteristic changes or customizations. EEG devices are mainly formed by the electrodes dedicated to signal conditioning and amplification, and for transferring data to the computer. As told before, the system has a total of 14 sensors or active channels, which correspond to the AF4, F8, F4, FC6, T8, P8, O2, O1, P7, T7, FC5, F3, F7, and AF3 positions. The positions correspond to the arrangement according to the international 10–20 standard (Klem et al., 1999). Figure 1 shows the positions of the electrodes in the international 10–20 system, as well as the location of the electrodes in the Emotiv Epoc+ helmet. Our approach includes the typical stages of this type of BCI system, namely, (1) signal acquisition, (2) data preprocessing, (3) feature extraction, and (4) classification. These stages are described below.

It is important to notice that the musical pieces used in this experimentation are in major mode. The results offered in this article would probably not apply in minor mode musical pieces in line with a series of studies (e.g., Husain et al., 2002; Knoferle et al., 2012).

### 2.2 Musical Experiment

**Figure 2:**
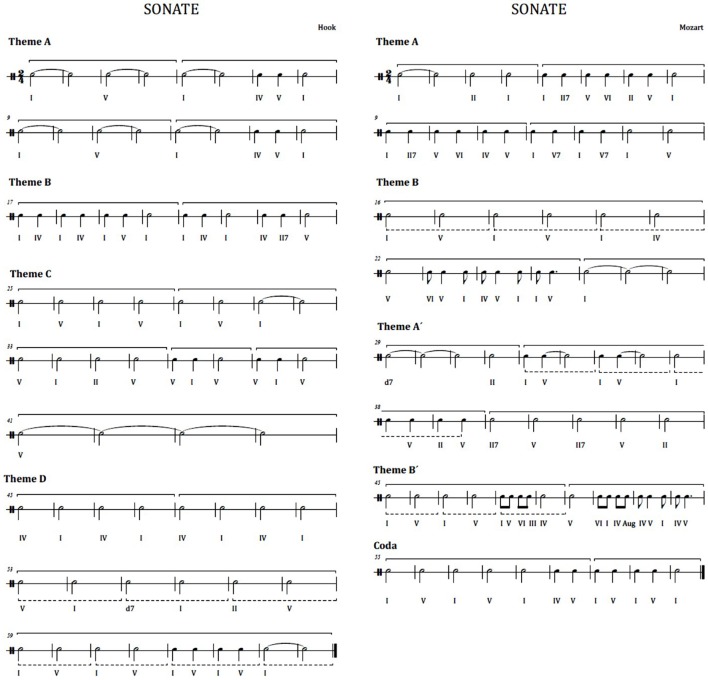
**Rhythmic harmonies**. Left: first movement of the Hook sonatina. Right: first movement of the Mozart sonatina.

This musical experiment exposes participants to listening the first movement of two different pieces in classical style, i.e., two sonatina movements. The first excerpt is the first movement of the sonatina in bipartite sonata form named “Sonatina for clarinet and piano in Bb Major” by Wolfgang Amadeus Mozart (see Figure 2 at the right). A bipartite sonata is a type of musical composition having a major theme (theme A), which is exposed in main tonality, followed by a theme B heard in the dominant, finishing the first section. In the second section, the first theme (theme A′) is exposed in the dominant and the secondary theme (theme B′) is presented in the tonic. In this case, the theme B is heard in the dominant because it adds a final coda, which is in the tone of the tonic. The second musical piece is the first movement of the “Sonata No. 1 for trumpet and piano in Eb Major” in rondo form, by James Hook (see Figure 2 at the left). Rondo form is a musical form based on the repetition of the same theme. The main theme reappears and alternates with different secondary themes. In this case, we have the ABACAD form (theme A followed by B, back to the theme A followed by the theme C, newly theme A, and finally theme D). Both sonatas are performed in a version for clarinet and piano, so that the change of instrumental timbre does not affect the listener. Moreover, both musical pieces are played at a same volume.

Electrophysiological data are captured to see the listener’s response to the phrase rhythm while listening to the music pieces. The physiological data from listeners are considered for evaluation of the musical experiment. In short, this experiment aims to measure the degree of activation using EEG in the electrophysiological response to the principle of repetition and contrast marked by the musical form, related to phrase rhythm. Therefore, we study Mozart’s music movement in bipartite form versus Hook’s rondo form piece.

### 2.3 Methodology

**Figure 3:**
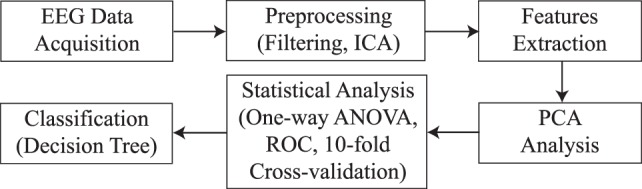
**Steps followed in the methodology**.

The methodology followed in this work is represented in Figure 3. First of all, EEG signals are acquired by means of the Emotiv EPOC+. Then, these signals are preprocessed by applying filters and artifact removal techniques. After that, frequency features are extracted for each EEG channel. Principal Component Analysis (PCA) is applied to reduce the number of features in further analysis. Then, statistical significance is calculated by a one-way ANOVA test and 10-fold cross-validation. Finally, a decision tree model is used for classifying the samples into the two groups of study.

#### 2.3.1 Data Preprocessing

As most biomedical signals appear as weak in an environment that is filled with many other signals, it is not a simple task to obtain relevant information from brain activity. Artifacts resulting from eye blinks or other muscular movements make signals more noisy and difficult to interpret. At this stage, different types of filter designs, covering a wide range of approaches, and algorithms to remove unwanted artifacts from the raw signal must be are used. In this work, the recorded EEG signals are preprocessed using advanced signal processing techniques (Koelstra et al., 2012). The baseline of each EEG channel is removed by applying a forward/backward filtering approach. The equation that describes the output *y*[*n*] of the digital filters used in this work is

(1) $$\begin{array}{ll} y[n] & =b_{0}\cdot x[n]+b_{1}\cdot x[n-1]+\ldots +b_{N}\cdot x[n-N]\\ & =\sum_{i=0}^{N}\;b_{i}\cdot x[n-i], \end{array}$$

where *x*[*n*] is the input signal, *N* is the filter order, and *b_i_* are amplitude coefficients of the impulse response at instant *i*.

Apart from the electrical information of the brain, EEG signals also contain artifacts produced by other co-occurring physiological signals. Technical artifacts, similar to electrode-pop peaks, may also appear (Barlow, 1986). All these artifacts can be removed by means of a blind source separation technique, such as independent component analysis (ICA) (Sanei, 2013). Artifacts are considered independent of the electrical activity of the brain, which makes ICA a suitable methodology to remove artifacts and maintain only the information related to neural activity.

Other types of artifacts cannot be eliminated by using ICA decomposition. It is the case of high-amplitude noise or interferences, mainly provoked by body movements or a poor contact of the electrodes over the scalp (Goncharova et al., 2003). To eliminate these artifacts, bad channels are usually removed and reconstructed by interpolating adjacent channels. Nevertheless, the low spatial density of the signals registered with Emotiv EPOC+ (only 14 channels) does not allow to apply this methodology. Instead, an adaptive filter should be used to reduce noise in EEG signals (Reddy and Narava, 2013).

#### 2.3.2 Feature Extraction

At this stage, EEG signals have been filtered and freed of artifacts. Thus, several analysis techniques are applied to extract the signals’ main features. Feature extraction allows to obtain certain specific characteristics of the acquired signals, which are useful for discriminating between different mental states. In the present work, the power spectral value of each EEG channel is estimated to obtain how power variance distributes as a function of frequency. Indeed, EEG is typically described in terms of rhythmic activity, where the frequency band is divided into different bandwidths (Michel et al., 1992). More concretely, the EEG spectrum is commonly separated into four different bands: *theta* (4–7 Hz), *alpha* (8–15 Hz), *beta* (16–31 Hz), and *gamma* (32–45 Hz). Each bandwidth is related with some kind of biological activity (Tatum, 2014).

In this work, a non-parametric method based on the Fast Fourier Transform (FFT) is used. FFT is based on the Discrete Fourier Transform (DFT) algorithm, with the particularity of the simplicity of its algorithm and a high processing speed. The DFT algorithm can be described as follows:

(2) $$X_{k}=\sum_{n=0}^{N-1}\;x_{n}\cdot e^{-j2\pi kn/N},\;k=0,1,\ldots ,N-1,$$

where *X_n_* is a sequence of complex numbers and *N* is the length of the sequence.

When a signal is transformed into the frequency domain, its waveform is subdivided into bandwidths, following the aforementioned boundaries. Finally, the spectral power (SP) for each bandwidth is calculated such that

(3) $$SP=\sum_{n=1}^{N}\;{\left[X\right]}^{2},$$

where *X* corresponds to the signal’s sample in the frequency domain. It is worth noting that SP is computed for each frequency band and for each EEG channel, and also that each participant listens to both musical pieces. Therefore, the feature matrix is an *N* × *M* matrix sized 40 × 56, where *N* is the number of samples (20 participants listening to 2 music pieces) and *M* is the feature vector (SP computed over each of the 4 frequency bands and each of the 14 EEG channels).

#### 2.3.3 Classification and Statistical Analysis

It is time now to classify the input parameters into a set of different patterns or classes and to statistically analyze the classification performance. The success of classification is determined by the appropriate choice of the parameters characterizing the signal and the effectiveness of the selected classification algorithms. In this work, Shapiro–Wilk and Levene tests are used to verify that data are normal and homoscedastic. Moreover, statistical differences between the two groups under study are calculated by using a one-way ANOVA test. In this sense, a value of statistical significance *ρ* ≤ 0.05/14 = 0.00357 is considered to be sufficient. In addition, each feature’s individual ability to discriminate between both groups is evaluated by using a Receiver Operating Characteristic (ROC) curve. This curve is the result of representing Sensitivity (*Se*) in front of 1 − Specificity (*Sp*). The sensitivity is the relation between true positive (*TP*) and all real positive values; in other words, true positives plus false negatives (*FN*), as can be seen in the following equation:

(4) $$Se=\frac{TP}{TP+FN}.$$

On the other hand, specificity is the result of dividing the number of true negatives (*TN*) into the number of real negatives (true negatives plus false positives (*FP*)), as shown in the next formula:

(5) $$Sp=\frac{TN}{TN+FP}.$$

Finally, the accuracy (*Acc*) for the classification of samples into the corresponding groups is defined as the relation between the number of samples properly classified (true positives and true negatives) and the whole population:

(6) $$Acc=\frac{TP+TN}{TP+FP+TN+FN}.$$

It is important to highlight that a ten-fold stratified cross-validation is used in this analysis. The reason for stressing the data is to avoid the possibility of classification results to be highly dependent on the choice of a given training-test segmentation. More precisely, for each experiment, the entire database is separated into ten equally sized folds, such that each fold is a good representative of the data. Then, ten learning and test iterations are performed. At each iteration, nine folds are randomly used to train the system, while the remaining fold is used to test the performance. Furthermore, at each iteration, a threshold is obtained for maximizing the ROC performance. Such threshold is then used to assess the performance on the test set.

With the aim of improving the classification performance and exploring the possible relationships among the different EEG channels, as well as the frequency bands activated, different classifiers are tested. Specifically, decision tree, linear and quadratic discriminant analysis, logistic regression, linear support vector machine, and k-nearest neighbor methods are applied on the data. However, in this study, the tree-based model shows the highest correctness in discriminating among groups. Therefore, this model is finally used to classify the data. In this regard, it is worth noting that some rules are imposed to prevent tree overgrowth. Thus, each node stops when it contains only samples from one class, or a group of subjects with less than 20% of all observations. Moreover, the splitting criterion used to evaluate the goodness of the alternative splits for each node is based on the impurity-based Gini index.

Finally, given the high amount of relevant features, some methodology for selection process or dimensionality reduction has to be carried out. Indeed, one of the difficulties that are inherent in multivariate analysis is the quantity of information that has to be managed. Often, a variable might be measuring the same driving principle than another one in the system. However, in many systems, there exist exclusively a few forces or variables governing the behavior of the whole system. Therefore, it is possible to reduce the variables by taking advantage of redundant information. In short, it is possible to simplify the system by replacing a group of variables with a new single variable that is a linear combination of the original ones. Principal Component Analysis (PCA) is the methodology used in this work to perform dimension reduction. PCA is characterized by generating a new set of principal components, all of them orthogonal to each other, eliminating redundant information (Jolliffe, 2002).

## 3 Results

In the study, no significant differences are found when the spectral power (SP) is computed for each EEG channel and for bands *beta* and *gamma*. On the contrary, a number of channels result significant when *theta* and *alpha* bands are studied. Tables 1 and 2 show the statistical significances for all the relevant channels and for *theta* and *alpha* bands, respectively. As you may appreciate, most EEG channels show statistical differences between the two groups and for the two bands. It is worth noting that the same channels show some discriminant power in both bands, *theta* and *alpha*. They belong to several areas of the brain, including frontal, parietal, temporal, and occipital zones.

**Table 1:** **Statistical outcomes for all the relevant channels and for *theta* bands**.

Ch	*ρ*	*Se* (%)	*Sp* (%)	Acc (%)
AF3	0.0136	66.50	56.10	59.70
F7	0.231	67.70	64.50	65.60
F3	0.00806	55.60	70.00	65.10
FC5	**3.35 × 10^−9^**	55.30	70.80	65.50
T7	**2.27 × 10^−8^**	64.30	60.20	61.60
P7	**0.00085**	66.50	66.70	66.60
O1	0.11	69.50	62.20	64.70
O2	**1.30 × 10^−13^**	62.40	70.60	67.80
P8	**1.88 × 10^−16^**	68.80	62.60	64.70
T8	**1.53 × 10^−7^**	65.40	59.50	61.50
FC6	**6.71 × 10^−12^**	66.20	55.80	59.30
F4	**2.15 × 10^−14^**	66.90	66.70	66.80
F8	0.00321	72.90	54.80	61.00
AF4	**6.81 × 10^−6^**	64.30	61.20	62.30

*First column: EEG channel. Second column: statistical significance; values in bold demonstrate statistical significance. Third column: sensitivity. Fourth column: specificity. Fifth column: global accuracy*.

**Table 2:** **Statistical outcomes for all the relevant channels and for *alpha* bands**.

Ch	*ρ*	*Se* (%)	*Sp* (%)	Acc (%)
AF3	**4.20 × 10^−11^**	64.70	63.90	64.20
F7	0.143	66.50	65.50	65.90
F3	**1.06 × 10^−5^**	65.40	67.30	66.60
FC5	**7.27 × 10^−16^**	61.70	65.70	64.30
T7	**2.48 × 10^−15^**	55.30	70.60	65.30
P7	**2.44 × 10^−16^**	57.50	65.10	62.50
O1	0.082	68.00	61.80	63.90
O2	**3.45 × 10^−9^**	57.50	69.20	65.20
P8	**3.04 × 10^−15^**	64.30	72.10	69.40
T8	**1.25 × 10^−7^**	68.00	61.20	63.50
FC6	**1.25 × 10^−14^**	63.50	57.10	59.30
F4	**3.39 × 10^−20^**	62.80	69.60	67.30
F8	**2.49 × 10^−12^**	75.20	51.50	59.60
AF4	**6.64 × 10^−12^**	53.80	74.10	67.10

*First column: EEG channel. Second column: statistical significance; values in bold demonstrate statistical significance. Third column: sensitivity. Fourth column: specificity. Fifth column: global accuracy*.

From a statistical point of view, the most remarkable differences between the groups are provided by parietal channel P8 and frontal channel F4 in the *theta* band. The discriminatory power reached by occipital channel O2 is also remarkable. Similarly, F4 is the most relevant channel in the *alpha* band. In addition, the symmetrically spaced parietal channels P7 and P8, as well as the frontal–central channels FC5 and FC6 reach comparable significance in this band. Finally, again T7 shows an important significance in the *alpha* band.

Moreover, the single discriminatory ability for each channel can be observed in Table 1 for *theta* band. It is important to remark that a 10 − *k*-fold approach is used to perform the analysis. Moreover, the analysis is repeated 10 times, so that the final sensitivity, specificity, and global accuracy correspond to the averaged value of these iterations. The O2 channel reports the highest discriminatory power in *theta* band, achieving a correctness of 67.80%. On the contrary, the FC6 channel achieves a lowest global accuracy of 59.70%. Regarding the frontal area, all EEG channels obtained a poor performance in the classification. Thus, F7 reports no statistical significance. Similarly, AF3, AF4, and F8 achieve global accuracies that range from 59.70 to 62.30%. Finally, the central channel FC5 and parietal channels P7 and P8 achieve a notable performance with global precisions of 65.50, 64.70, and 66.70%, respectively. With respect to the *alpha* band (see Table 2), P8 achieves the highest performance in the classification with a 69.40% of correctness. On the contrary, and similar to the previous case, FC6 reports the lowest global accuracy with 59.30%. The rest of the channels achieve comparable performances, ranging from 62.50 to 67.30% of correctness.

**Figure 4:**
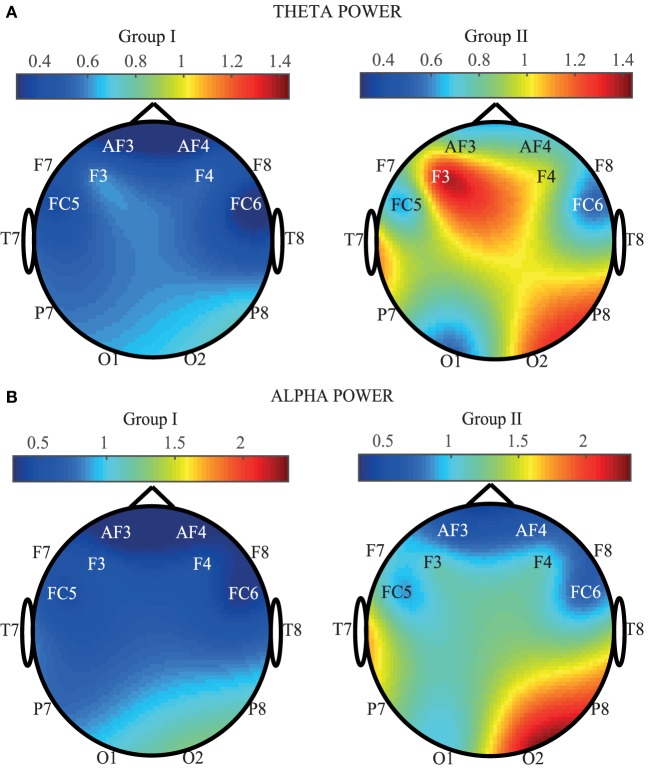
**Comparison of average SP for each EEG channel and for the two groups (Group I: Hook sonata, Group II: Mozart sonata)**. **(A)** In the *theta* band. **(B)** In the *alpha* band.

In order to provide a spatial view of brain activation, Figure 4 provides EEG maps containing the average of SP calculated for each channel and for the two groups. More concretely, in Figure 4A, the *theta* power average (in ×10^7^) for each EEG channel is shown. As it can be appreciated, an increase of the SP is observed in certain areas of the EEG for the Mozart sonata. On the contrary, a generalized decrease of power is observed when people listen to the Hook sonata. Frontal, right parietal, and right occipital areas are the most active zones. Indeed, F3, F4, P8, O2, and T7 show the highest increase in power. These results are in concordance with the outcomes obtained throughout the one-way ANOVA test. On the other hand, Figure 4B represents the averaged *alpha* power computed for each EEG. In a similar manner to the previous case, an increase of the SP is observed in group II, especially in the frontal, right parietal, and right occipital channels. On the contrary, a generalized decrease of the SP can be appreciated in group I. These results are in agreement with the reported statistical significance, as all central and parietal channels report a comparable discriminatory power. Nevertheless, it is important to highlight that P8 and O2 obtain the highest power increase in the *alpha* power.

**Figure 5:**
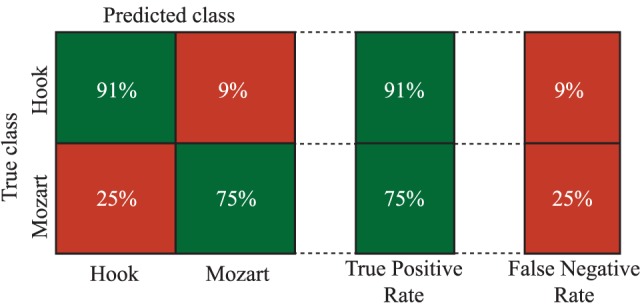
**Confusion matrix**.

In order to study the possible relationships among EEG channels and bands, a tree-based model classifier is used. Nevertheless, given the high amount of available information, the features are reduced using PCA, according to the methodology described in Section 2.3.3. In this regard, components are included in the analysis until the sum of the variances reaches 95%. More concretely, 10 out of 21 components are chosen. It is worth noting that only relevant EEG channels from *theta* and *alpha* bands are included in the PCA analysis (see Tables 1 and 2). Finally, the components are used to feed the tree classifier. The global accuracy obtained with this methodology reaches 85.5% correctness. This involves an increase in the global performance of more than 15% regarding the best single parameter achieved by P8 in the *theta* band. In Figure 5, more detailed information about the sensitivity and specificity obtained with this model is offered by means of a confusion matrix. It is important to remark the ability of the algorithm in classifying correctly the Hook sonata, with 91% of probability, against the sensitivity reported by F8 in the ROC analysis.

## 4 Discussion

The results observed in our experimentation show statistical differences between the musical pieces (bipartite vs. rondo) for *theta* and *alpha* bands. These studies are dedicated to provide information on the neural correlates of phrase rhythm through observing brain activity by EEG signals. The results offer a view on the changes that occur in the emotional state of a music listener when listening to two different sonatas. These results are in line with other recent works that are described next.

In a study with three excerpts of instrumental classical and rock music (Schmidt and Hanslmayr, 2009), the authors show that resting EEG *alpha* activity over frontal electrode sites predicts evaluations of affective musical stimuli. Left-active individuals with relatively greater *alpha* power over right frontal electrode sites rated all stimuli as more positive than right-active individuals. These results indicate that frontal *alpha*-asymmetry reflects interindividual differences in affective response tendencies to emotional musical stimuli. Also, a very recent study with two ragas (Hindustani music) chosen for analysis were “Chayanat” (romantic/joy) and “Darbari Kannada” (pathos/sorrow) (Banerjee et al., 2016). EEG signals were acquired at the frontal (F3/F4) lobes of the brain while listening to music at three experimental conditions (rest, with music, and without music). The finding shows that arousal-based activities were enhanced while listening to Hindustani music of contrasting emotions (romantic/sorrow) for all the subjects in case of *alpha* frequency band. In the same line, three music clips, taken from Mozart’s sonata (MS)-K. 448, Brahms’ Hungarian dance no. 5 (BHD), and a simplified version of the theme taken from Haydn’s symphony no. 94 played by a computer synthesizer (HS), of 6-s duration, which were repeated in random succession 30 times each, were analyzed, and the conclusion was that the power spectra revealed a significant difference only in the lower −1 *alpha* band (7.17–9.16 Hz) (Jausovec and Habe, 2003). Again, the *alpha* band reveals to be discriminant in all these studies, as in our experiment.

It is worth noting that our experiment reveals that the left frontal and left temporal channels show important differences between the groups under study. These results are in agreement with the existing literature, regarding the induction of emotions through music. Furthermore, the activation of the right occipital area is remarkable, more specifically in the *alpha* band.

To conclude, this paper has introduced our experimentation toward understanding the neural correlates of phrase rhythm. Our interest in this kind of approaches dedicated to music-evoked emotions is related to a research that aims to find solutions capable of improving the quality of life and care of aging adults who can or want to keep living at home. We are convinced that certain musical parameters related to rhythm are useful to guide the aging adult’s emotional state to a pleasant mood. In this experiment, phrase rhythm is studied through forcing brain activation. By means of a BCI used as a simple EEG, the neural correlates have been studied in the frequency domain. The power spectral value of each EEG channel has been estimated to obtain how power variance distributes as a function of frequency.

The musical experiment has offered the listener two sonatina movements. The first of them is the first movement of the sonatina in bipartite sonata form named “Sonatina for clarinet and piano in Bb Major” by Mozart. The second one is the first movement of the “Sonata No. 1 for trumpet and piano in Eb Major” in rondo form, by Hook. Both sonatas provide sufficient differences in phrase rhythm to be studied in relation the activity in certain brain areas. More concretely, we have studied the electrophysiological response to the principle of repetition and contrast marked by both musical forms. The results of processing the acquired signals are in line with previous studies that use different musical parameters to induce emotions. Indeed, our experiment shows statistical differences in *theta* and *alpha* bands between two classical sonatas with variations in their phrase rhythm. Nonetheless, it is our intention to replicate the experiment with a 64-channel EEG in a future work. This new experiment will be able to validate the results gotten in this work, as well as to assess the precision of a BCI used as an EEG in the frequency domain.

## Ethics Statement

The present study is carried out in accordance with the ethical standards and the approval of the committee on human experimentation from Universidad de Castilla-La Mancha with written informed consent from all subjects in accordance with the Declaration of Helsinki.

## Author Contributions

All the authors contributed to the different phases of the research and to the writing of this paper.

## Conflict of Interest Statement

The authors declare that the research was conducted in the absence of any commercial or financial relationships that could be construed as a potential conflict of interest.
